# The microbial pathology of maternal perinatal sepsis: A single-institution retrospective five-year review

**DOI:** 10.1371/journal.pone.0295210

**Published:** 2023-12-27

**Authors:** James Powell, Clare M. Crowley, Brid Minihan, Mendinaro Imcha, Nuala H. O’Connell, Roy K. Philip, Colum P. Dunne

**Affiliations:** 1 Department of Microbiology, University Hospital Limerick, Limerick, Ireland; 2 School of Medicine and Centre for Interventions in Infection, Inflammation, and Immunity (4i), University of Limerick, Limerick, Ireland; 3 Department of Obstetrics and Gynaecology, University Maternity Hospital Limerick, Limerick, Ireland; 4 Division of Neonatology, Department of Paediatrics, University Maternity Hospital Limerick, Limerick, Ireland; 5 University of Limerick School of Medicine, Limerick, Ireland; Universita Politecnica delle Marche, ITALY

## Abstract

**Introduction:**

Greater than half of in-hospital maternal deaths are caused by sepsis, a condition that occurs when infection exceeds local tissue containment and results in organ dysfunction. Determining the source of infection can be challenging. Microbiological cultures of the uterine cavity are often difficult to obtain, so antimicrobial susceptibility results may not be available to guide treatment. The aim of this retrospective study was to assess the potential clinical value of microbiology samples used in the maternal “septic screen” of patients in an Irish maternity hospital.

**Methods:**

A review was completed of all maternal “septic screen” (i.e., high vaginal swabs, placenta swabs, blood cultures, throat swabs and urine samples) microbiology results from July 2016 to December 2021.

**Results:**

In the relevant period, 845 patients were subject to a “septic screen”, of whom 430 also had a placental swab collected. These 430 patients comprise our study population. 2% of blood cultures yielded potential pathogens, compared with 37%, 33%, 9% and 7% respectively for placental swabs, high vaginal swabs, throat swabs and urine specimens. 95% of blood cultures were sterile, compared with 52%, 0%, 0% and 53% respectively for placental swabs, high vaginal swabs, throat swabs and urine specimens.

**Conclusion:**

Of the five microbiological specimen types examined, placental swabs yielded the highest number of potential pathogens. Our results suggest that placental swabs are useful specimens for detecting potential pathogens from the uterine cavity, the most common source of perinatal infections.

## Introduction

Globally, approximately 75,000 pregnant women die annually due to sepsis, with the majority of these deaths occurring in low income countries [1]. Ireland has a maternal mortality rate of five cases per 100,000 live births, equal to that seen in Western Europe and lower than that of North America (18), Latin America and the Caribbean (74), East Asia and Pacific (72), South Asia (163) and Sub-Saharan Africa (533) [2]. “Near misses” resulting in severe maternal morbidity occur ten [3] to 50 [4] times more frequently than maternal mortalities. Greater than 10% of maternal deaths [5] and more than half of in-hospital maternal deaths [6] are caused by sepsis, yet despite being the third most common cause of maternal death, maternal sepsis receives less attention than other pathologies [5].

Sepsis occurs when an infection exceeds local tissue containment and induces a series of dysregulated physiological responses that result in organ dysfunction [7]. In 2017, the World Health Organisation (WHO) provided a consensus definition for maternal sepsis as “a life threatening condition defined as organ dysfunction resulting from infection during pregnancy, childbirth, post-abortion or post-partum period” [8]. Sepsis usually presents itself first as Systemic Inflammatory Response Syndrome (SIRS), which is “an exaggerated defense response of the body to a noxious stressor”, in this case an infection [9]. As the placental bed is highly vascular, maternal bacteraemia leading to sepsis can develop quickly [10]. In a UK study of Group A streptococcal infection, 50% of patients had severe sepsis within 2 hours (75% in 9 hours) of the first signs of a systemic inflammatory response [11]. The early recognition, diagnosis and management of maternal sepsis leads to better maternal and foetal outcomes, with a linear relationship reported between mortality and hourly antimicrobial treatment delay [4].

Determining the source of maternal infection can be difficult for clinicians. Chorioamnionitis (“Triple I” [12]) and endometritis are the two most prevalent maternal infections (24% and 23%, respectively) [13]. Chorioamnionitis is acute inflammation of the chorion and amnion (foetal membrane layers), most commonly resulting from polymicrobial infection ascending from the vaginal canal into the uterine cavity in the setting of premature or prolonged rupture of membranes. Triple I was proposed to replace the term chorioamnionitis since intrauterine infection may also involve the amniotic fluid, foetus, umbilical cord or placenta in addition to the foetal placental membranes [14]. Endometritis is considered a postpartum infection of the uterine lining/cavity typically caused by polymicrobial infection. Risk factors for infection include preterm prelabour rupture of membranes, manual placental removal and suspected retention of products of conception [15, 16]. Diagnosis of triple I is often suspected clinically but not confirmed until placental histology results are available in the postpartum period [17]. Microbiological evidence can be difficult to obtain as microbiological cultures of the uterine cavity are often contaminated by vaginal flora, which may hinder detection of infecting agents and appropriate choice of antimicrobial agent for treatment [10]. Screening for Group B Streptococcus (*Streptococcus agalactiae* or GBS) remains a controversial topic regarding the optimal strategy to employ [18]. Screening in our hospital typically involves the analysis of first-trimester urine specimens. Vaginal swabs are infrequently conducted for asymptomatic patients, but GBS screening is performed when received. On occasion, vagino-rectal swabs are also submitted for analysis.

In an international study across 52 countries, more than one third of women who were diagnosed with an intrauterine infection experienced additional complications including severe maternal outcomes [6]. Blood cultures may be negative in 28–49% of septic patients [19], yet all-cause mortality is not statistically different between culture-negative and culture-positive sepsis patients [20]. Organism identification only occurs in 50% of maternal sepsis cases [21], and diagnosis can be hampered by physiological pregnancy-related changes. For instance, white blood cell count and C-reactive protein typically increase during pregnancy, particularly during labour and postpartum, further hindering sepsis recognition [22]. Rapid identification of the causative pathogen facilitates appropriate antibiotic treatment, which is known to improves survival rates, patient outcomes, reduces hospital length of stay and overall hospital expenditures [23]. The inappropriate use of antimicrobials has been linked with patient harm either directly, by precipitating *Clostridioides difficile* infection or the development of antimicrobial resistance [24].

In our hospital group, septic screens are completed in accordance with Irish national clinical guidelines. These are available as a guidance document [25], a review article [3] and a sepsis predisposition and recognition form [26]. They are updates of previous versions [27, 28]. The three most relevant international published guidelines on maternal sepsis are provided by the Royal College of Obstetricians and Gynaecologists [29], The Society for Maternal-Fetal Medicine [30] and the Society of Obstetric Medicine of Australia and New Zealand [31]. The recommended specimen types to collect for microbiological testing are listed for each set of guidelines in S1 Table. However, these guidelines do not provide a rationale for the utilization of placental swab cultures or describe potential clinical benefits for testing any of the listed specimens.

Placental swab cultures have been reported as useful in guiding antimicrobial therapy for the neonate [32–34], although this is not accepted universally [35, 36]. There are limited data available describing the merits of placental swab cultures for the investigation of maternal sepsis, despite their inclusion in international guidelines [29, 31]; and there is no uniformity in collection procedures for maternity hospitals nationally. The collection of placental swabs is not standardised in our institution; they are collected at the clinicians’ discretion, when chorioamnionitis is suspected clinically, foetal death, maternal pyrexia in labour, prolonged rupture of membranes (PROM) or preterm prelabour rupture of membranes (PPROM) or if foul smelling liquor is noted. The aim of this study is to compare the microbiology results of samples collected as part of maternal septic screens. The provision of this data describing the specimen collection process and stratification of specimen results will be clinically useful when devising future maternal sepsis guidelines. This assessment also considered other laboratory specimens collected as part of the septic screen that include blood cultures, high vaginal swabs (HVS), urine specimens and throat swabs. Respiratory specimens and cerebrospinal fluid (CSF) may also be collected as dictated by the clinical situation. All of the investigations discussed in this study are culture based tests which are accessible across a spectrum of income settings and countries. Consequently, the integration of the findings from this study into clinical practice could potentially have a wide application in investigation and management of maternal sepsis, potentially mitigating mortality associated with this severe condition.

## Methods

For background, the Department of Clinical Microbiology at University Hospital Limerick provides a centralised microbiology service for the six acute hospital sites of the UL Hospitals Group (867 current beds) and community healthcare facilities catering for a population of 473,269 people in the Mid-West of Ireland. University Maternity Hospital Limerick (UMHL) is the only maternity hospital in the region, with circa 4,500 live births per annum. Previous related research from our institution includes a neonatal case study and literature review highlighting the merits of placental swab cultures [32], a number of neonatal [37, 38] and other [39, 40] outbreak reports, and a retrospective review of the time–to- positivity of neonatal blood cultures [41].

All “septic screen” microbiology results from July 2016 to December 2021 were extracted from the Laboratory Information Management System (“LIMS”, iLab, DXC Technologies). This included High Vaginal Swabs (HVSs), placenta swabs (almost exclusively one swab from the maternal side and one from the foetal side), blood cultures, throat swabs, urine samples, respiratory specimens and cerebrospinal fluid (CSF). Blood cultures were tested using the BacT/ALERT® 3D system (BioMérieux PLC, Marcy-l’Étoile, France), all positive blood cultures and swab cultures were cultured on 90mm Petri-dish pre-poured commercially supplied agar from BioMérieux PLC or LIP Diagnostics, Fannin Ltd, Galway, Ireland. All microbiology specimens are tested within a 48 hour timeframe upon receipt. More than 75% of samples are analysed within 12 hours of receival and are then refrigerated if they are held outside that timeframe. Specimens received in the laboratory more than 48 hours after collection from the patient are rejected for analysis as they are considered too old. Transport times and analysis times are continuously monitored as “key performance indicators” of the laboratory. Inclusion criteria included patients who had vaginal swab requests with “sepsis” or “query sepsis” on the accompanying request form (i.e., a septic screen workup was requested), and also had placental swabs collected. Septic screens are requested if a patient displays any of the following symptoms: Pyrexia, tachycardia, tachypnoea, hypotension, oliguria, confusion or altered mental state.

The inclusion criterion of patients with placental swabs in our study ensured non-perinatal patients were excluded. The delivery date for each patient was identified from the collection date of the placental swabs (which are collected at delivery). Specimens collected within one week of the delivery date were eligible for inclusion. A comparison group of placenta swabs from 1,318 patients that did not meet the inclusion criteria of the study, i.e. did not have a “septic screen” were also analysed. These patients had placenta swabs collected (see S1 File for clinical indications to collect placenta swabs in our hospital) but were not highlighted as “query sepsis” on the specimen request form. The LIMS was also examined for records of GBS screening tests and antimicrobial susceptibility percentages. The Hospital In-Patient Enquiry database (HIPE) is a computerized system designed to capture the administrative, demographic and clinical data on all inpatient discharges in all publicly funded hospitals in Ireland. HIPE is considered a robust source for public health related research [42, 43]. The HIPE database was queried for relevant morbidity data for this study. Of note, there are no electronic patient records in our hospital.

By examining the study population over a long period (5½ years) we maximise the precision of the analysis and generalisation of results. The data were analysed and summary statistics were generated using Microsoft Excel 2016. Further statistical analysis was performed using IBM SPSS V.28.0.1.1. Post hoc analysis of chi-square contingency tables were performed as previously described [44].

This study was approved by the Research Ethics Committee of the University of Limerick Hospital Group, Limerick, Ireland (Ref 071/2022).

## Results

During the 5½ year study period there were a total of 23,080 deliveries in UMHL. 98% (22,624/23,080) were singleton and 2% (456/23,080) were multiple pregnancies. In total, 845/23,080 (3.7%) patients had a “septic screen” completed and, of those, 51% (430/845) also had a placental swab collected. This latter group of 430 patients represented our study cohort, ranging in age from 17–44 years (median 31 years, IQR 26–34 years). Five of the 430 patients required admission to the intensive care unit within one week of delivery.

Delivery information (from HIPE) was available for 410 (95%) patients; 15 patients had no HIPE details and insufficient HIPE information was recorded for five patients. Delivery demographics are described in Table 1. In total, 98% of single infants were delivered and 2% were multiple births. 91% of infants were delivered after 37 weeks gestation, and 96% of the total were liveborn.26% (106/410) of women had an episiotomy, and 2% (8/410) of women required manual removal of retained placenta. 7% (30/410) of patients were recorded as current smokers.

**Table 1:** Delivery details per gestation length.

Gestation	Singleton	Twins	Spontaneous Vaginal	Vacuum/ Forceps	Caesarean	Liveborn	Stillborn	Total
< 20 Weeks	7	1	8	0	0	0	8	8
20–25 Weeks	4	1	5	0	0	1	4	5
26–33 Weeks	7	0	2	0	5	7	0	7
34–37 Weeks	15	3	6	1	11	18	0	18
> 37 Weeks	369	3	59	126	187	369	3	372
Total	402	8	80	127	203	395	15	410

### Bacteriology specimens

A summary of the findings of each of the specimens can be found in Table 2. Specifically:

**Table 2:** Pathogens identified per specimen type.

	Blood	HVS	Urine	Throat	Placenta
	n (%^α^)	n (%^α^)	n (%^α^)	n (%^α^)	n (%^α^)
Anaerobe	1 (0.0%)	77 (17.9%)	N/A	5 (1.5%)	62 (14.4%)
Beta Haemolytic Strep.Gp.B	2 (0.5%)	56 (13.0%)	7 (1.9%)	0 (0.0%)	34 (7.9%)
Other Beta Haemolytic Strep	1 (0.2%)	2 (0.5%)	1 (0.3%)	4 (1.2%)	3 (0.7%)
Other *Streptococcus*	3 (0.7%)	8 (1.9%)	3 (0.8%)	19 (5.7%)	35 (8.1%)
*Escherichia coli*	1 (0.2%)	3 (0.7%)	10 (2.7%)	0 (0.0%)	16 (3.7%)
Other Enterobacterale	0 (0.0%)	8 (1.9%)	2 (0.5%)	1 (0.3%)	51 (11.9%)
*Haemophilus* sp	0 (0.0%)	N/A	0 (0.0%)	N/A	3 (0.7%)
*Acinetobacter* species	0 (0.0%)	0 (0.0%)	0 (0.0%)	N/A	2 (0.5%)
Other Gram negative	1 (0.2%)	0 (0.0%)	0 (0.0%)	0 (0.0%)	3 (0.7%)
*Enterococcus* species	0 (0.0%)	5 (1.2%)	5 (1.3%)	0 (0.0%)	18 (4.2%)
*Staphylococcus aureus*	0 (0.0%)	2 (0.5%)	0 (0.0%)	1 (0.3%)	3 (0.7%)
*Staphylococcus lugdunensis*	0 (0.0%)	0 (0.0%)	0 (0.0%)	0 (0.0%)	3 (0.7%)
*Actinomyces* Species	0 (0.0%)	0 (0.0%)	N/A	0 (0.0%)	2 (0.5%)
Total Samples With Pathogens^β^	9	142	27	30	160
% With Pathogens	2.1%	33.0%	7.2%	9.0%	37.2%
*Lactobacillus* species^γ^	1 (0.2%)	N/A	1 (0.3%)	N/A	18 (4.2%)
Yeast^γ^	0 (0.0%)	86 (20.0%)	7 (1.9%)	18 (5.4%)	22 (5.1%)
Mixed Undifferentiated Growth^γ^	0 (0.0%)	0 (0.0%)	50 (13.3%)	0 (0.0%)	0 (0.0%)
Total Commensals^¥^^γ^	12 (2.8%)	233 (54.2%)	56 (14.9%)	290 (86.6%)	30 (7.0%)
No Growth	403 (95.0%)	0 (0.0%)	201 (53.3%)	0 (0.0%)	224 (52.1%)
No. Samples Tested	424	430	377	335	430

^*γ*^Specimens containing these organisms only.

^*¥*^ *Lactobacillus* species, Coagulase Negative *Staphylococci*, mixed undifferentiated growth, yeast, ^*α*^Percentage of total number of specimens tested. ^*β*^The total number of samples with pathogens is less than the sum of individual pathogens due to some samples having more than one pathogen detected.

464 sets of blood cultures were collected from 424 patients, i.e., 99% (424/430) of the patients had blood cultures collected, and of those, 94% had one set (comprising of an aerobic bottle and an anaerobic bottle), two patients with two sets each, and ten patients with more than two sets. 95% (443/464) of the blood culture sets were sterile. Twenty-one patients had positive cultures of which 12 sets yielded organisms deemed to be skin contaminants (coagulase negative *Staphylococcus* species (n = 8), *Micrococcus* species (n = 2), *Propionibacterium* species (n = 1) and *Lactobacillus* species (n = 1)), resulting in a contamination rate of 2.6% (12/464). Nine patients yielded organisms deemed to be potential pathogens, see Table 3 for details of those patients and the detection of those organisms from the other specimens in our study.

**Table 3:** Significant isolates from blood cultures and their detection in other specimens from these patients.

					Placental Swabs	
	Blood	Vagina Swab	Urine	Throat	Maternal	Foetal
Patient 1	*Streptococcus viridans*	No Pathogens	Sterile	No Sample	☑	☑
Patient 2	*Streptococccus agalactiae*	☑	No Sample	No Sample	☑	☑
Patient 3	*Streptococcus oralis*	Other^1^	Sterile	No Pathogens	Other^1^	Other^1^
Patient 4	*Peptoniphilus harei*	No Pathogens	No Pathogens	No Pathogens	Other^2^	No Pathogens
Patient 5	*Streptococcus agalactiae*	☑	No Pathogens	No Pathogens	☑	☑ Other^3^
Patient 6	*Pseudomonas aeruginosa*	No Pathogens	Sterile	No Pathogens	Sterile	Sterile
Patient 7	*Escherichia coli*	☑	Sterile	No Sample	☑	☑
Patient 8	*Streptococcus anginosus*	No Pathogens	No Pathogens	No Pathogens	Sterile	Sterile
Patient 9	*Streptococcus agalactiae*	☑	Sterile	No Pathogens	☑	☑

^*1*^*Anaerobe*, ^*2*^Coliform, *Enterococcus* species, mixed anaerobes, ^*3*^Coliform.

From these nine patients with significant organisms detected in blood cultures, five patients had the same organisms identified from corresponding placental swabs (55.6% sensitivity), and four patients from corresponding vaginal swabs (44.4% sensitivity). No significant isolates which were detected in blood cultures were detected in corresponding urine samples or throat swabs.

The low positivity rate (5% positivity) of blood cultures in this study was further analysed for the period 2020 to 2021. Data from our study cohort (“query sepsis” patients with placenta swabs collected, n = 202) were compared with the full cohort of all obstetric patients in our hospital (n = 8,206). The study cohort were more likely to have blood cultures collected (99%, 199/202) than the generalised sample (8%, 630/8206), but the positivity rate of the study group (5%, 9/199) was lower than that of the generalised group (6%, 37/630). The contamination rates were equal (3% contamination) for both groups. For comparison, the positivity rate for the non-obstetric cohort in our region in this period was 15% (2,926/19,838) for all patients and 10% (125/1,279) when controlled for age (19–44 years) and gender (female). The contamination rates for the non-obstetric group were higher (7% and 5% respectively) than the obstetric group (3%).

97% (416/430) of patients had a pair of placental swabs collected, one swab from the maternal and foetal side of the membrane respectively. The remaining swabs were unpaired or undifferentiated. 86% (357/416) of the paired specimens had similar results from both sides and 14% (59/416) had a significant organism detected from one side of the membrane only, predominantly from the foetal side (80%, 47/59). Taking each set of swabs as a single test, 224 sets (52%, 224/430) were sterile, and 41 (9.5%) yielded non-pathogenic organisms (coagulase negative staphylococci, *Corynebacterium* species, *Micrococcus* species, yeast). Anaerobes were the most prevalent potential pathogens (15%, 63/430), rarely as monomicrobial cultures (4%, 17/430), or mixed anaerobes with no other organisms (1%, 5/430). Most anaerobes were detected in a mixed growth with coliforms (12%, 51/430) and/or streptococci (3%, 13/430). *Bacteroides* species were the most frequently detected anaerobe (n = 11), followed by *Gardnerella vaginalis* (n = 8), however most anaerobes were not identified (particularly when mixed) and were reported simply as “anaerobe”. Beta Haemolytic *Streptococcus* group B (*Streptococcus agalactiae* or GBS) was the most commonly detected individual organism in 8% (34/430) of samples. GBS was also the most common monomicrobial culture (n = 23).

The results of organisms identified from placental swabs were classified according to mode of delivery: Placental swabs collected at caesarean section were more likely to be sterile (62%, 126/203) than swabs obtained at operative vaginal delivery (41%, 52/127) and spontaneous vaginal delivery (37.5%, 30/80). This finding indicates that placental bacterial contamination may occur during vaginal deliveries, particularly when superficial swabs of the placental surface are collected. This is substantiated by the profile of microorganisms detected, with *E*. *coli*, other enterobacterales and anaerobes detected in 7%, 18% and 24% of placental swabs from vaginal deliveries, compared with 0%, 2% and 6% of swabs from casesarean sections. However, only 37.5% (95/253) of significant organisms identified from placental swabs had the same organism identified on corresponding vaginal swabs, indicating that contamination was unlikely to have occurred in specimens where the pathogen detected from placental swabs were not detected in the corresponding vaginal swab (62.5%, 158/253). Some variation was found between different bacteria and their detection from these two sites: 12.5% (2/16) of patients with *E*. *coli* on placental swabs also had this organism detected on corresponding vaginal swabs. Conversely, 82.4% (28/34) of GBS positive samples were also positive on vaginal swabs.

**Fig 1:**
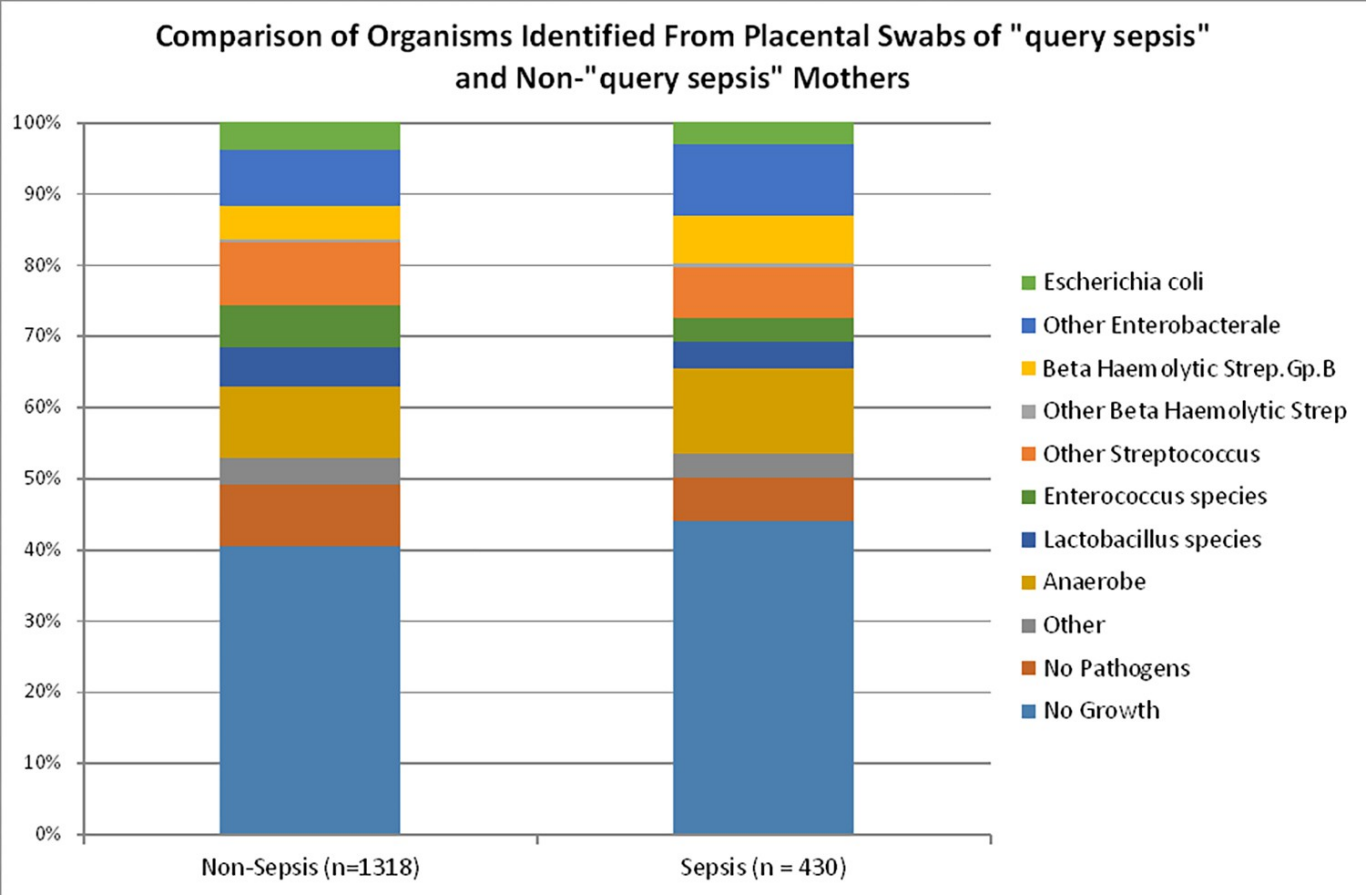
Organisms identified from placental swabs of “query sepsis” patients and non-“query sepsis” patients.

The results of organisms identified from the 430 placental swabs labelled as “query sepsis” were compared with 1,318 placental swabs from non-“query sepsis” sample during the same study period. See Fig 1 for a graphical representation of the main pathogens identified. The placental swab cultures from the “query sepsis” group had a greater proportion of sterile samples, GBS, other beta haemolytic Streptococci, enterobacterales (except *E*. *coli*) and anaerobes. The placental swabs from non-“query sepsis” group had proportionally more commensals, lactobacilli, enterococci, other streptococci and *E*. *coli*. These differences were not statistically significant when analysed using chi-square contingency-tables to produce adjusted standardized residuals: None of the Bonferroni-adjusted p-values that were produced were below the Bonferroni-adjusted significance threshold.

All 430 patients had a HVS collected (as per inclusion criteria of the study, but only 377 (88%) patients has a urine sample collected, and 335 (78%) of the 430 patients had a throat swab collected as part of the “septic screen”. No respiratory specimens (sputum, pleural fluid, bronchial washings) were collected, which is notable because the study period included almost two years of the COVID-19 pandemic period and the heightened respiratory vigilance associated with it. No cerebrospinal fluids were collected during the study period.

None of the HVS were sterile, 67% (288/430) had no pathogen detected and the most frequently detected potential pathogens from the remaining 142 patients were: Anaerobes (18%, n = 77), GBS (13%, n = 56) and *Escherichia coli* or other enterobacterales (2.5%, n = 11). Two patients yielded *Staphylococcus aureus*.

Of the 377 patients with urine specimens tested, 350 (93%) yielded either no growth or no significant growth (mixed undifferentiated growth, coagulase negative staphylococci, yeast). *Escherichia coli* was the most prevalent potential pathogen detected (10/377), followed by GBS (7/377). 91% (305/430) of the throat swab samples failed to detect a pathogen. *Streptococcus* species were the most commonly detected pathogen (7%, 23/335), predominantly *Streptococcus milleri* group (n = 19), followed by Beta Haemolytic *Streptococcus* other than *Streptococcus pyogenes* (Group A Strep, or GAS, n = 3). Five samples yielded anaerobes: *Staphylococcus aureus*, GAS and an enterobacterale were detected once each. This was the only detection of GAS from all of the specimens analysed in the study.

In total, 63 patients had GBS detected from a specimen in their “septic screen”, most commonly from HVS (n = 56; 25 from HVS alone) or placental swabs (n = 34; six from placental swabs alone). 94% (59/63) of this cohort of patients had GBS screening performed earlier in their pregnancy, either from urine collected in the first (n = 54) or third (n = 3) trimester,or vagino-rectal swabs taken near term (n = 2). The urine specimens had poor sensitivity: 32% (13/41) of first trimester and 33% (1/3) of third trimester urines were positive for GBS. Both vagino-rectal swabs cultured GBS, however.

Antimicrobial resistance is not prevalent in our hospital; susceptibility of our *E*. *coli* isolates to ampicillin, co-amoxiclav, gentamicin, pipercillin/tazobactam and meropenem for the study period were 41%, 79%, 93%, 96% and 100% respectively. The percentage of ESBL producers was 7% and carbapenemases were not detected. For *Staphylococcus aureus*, percentage susceptibility to flucloxacillin, clindamycin, tetracycline and vancomycin were 75%, 73%, 96% and 100% respectively. For GBS, clindamycin susceptibility was 65%, both penicillin and vancomycin were 100%. Antimicrobial prescribing guidelines for our hospital are available as S2 File.

The final 22 months (33%) occurred during the COVID-19 pandemic, therefore the patients were cross-checked for SARS-CoV-2 infection. 6% (24/430) patients tested positive for COVID-19 during the study period, but only one patient exhibited a positive result within one week of delivery, hence SARS-CoV-2 infections were not considered an influential factor on the results.

## Discussion

**Fig 2:**
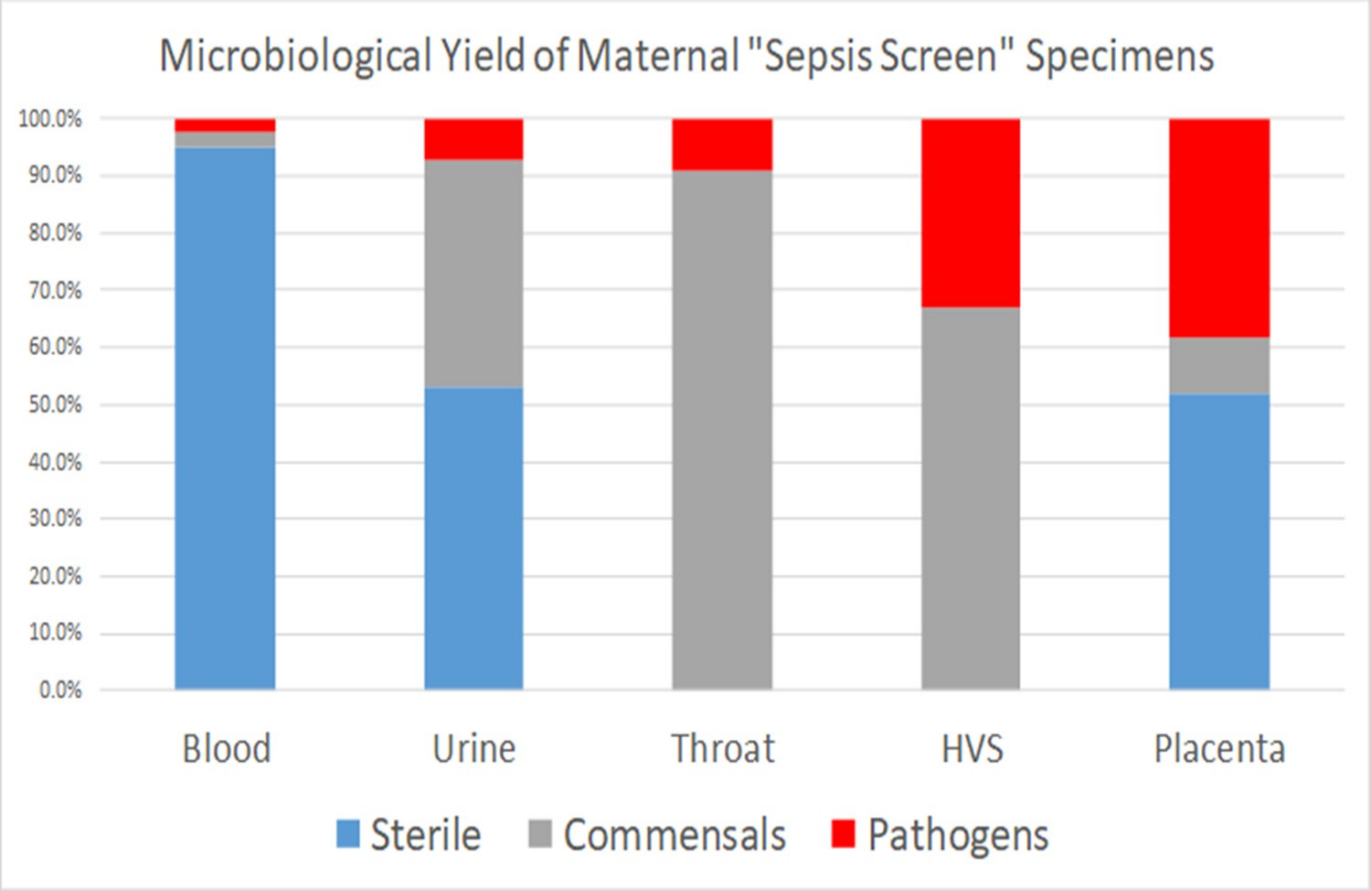
Microbiological yield of “septic screen” specimens.

The primary aim of this study was to compare the microbiology results of five different specimen types collected as part of maternal septic screens. Overall, 58% (250/430) of the subjects in our study had a significant pathogen detected across all of their “septic screen” microbiology specimens, comparable with 50% reported internationally [21]. From the five microbiology specimens examined, placental swabs yielded the highest number of potential pathogens (37%, 165/430) (Fig 2).

Anaerobes were the most common microorganisms cultured on both placental (n = 62) and high vaginal swabs (n = 77). Placental swabs detected the highest number of enterobacterales (n = 51 versus eight from vaginal swabs) and vaginal swabs detected the greatest level of *Streptococcus agalactiae* (n = 56 versus 34 from placental swabs). Given that 10–30% of women are colonised with *Streptococcus agalactiae* in the vagina or rectum [45], it is not surprising that high vaginal swabs detected a high yield of these organisms. The time difference between sampling of vaginal swabs and placental swabs was examined to assess its potential influence on the results. In total, 61% of the samples were collected on the same day, and 95% were collected within 48 hours of each other. Consequently, the time difference between sampling was unlikely to have imparted a significant impact on the results. While HVS have a high sensitivity in predicting colonisation of the lower genital tract, the positive predictive value for detecting intrauterine infection is low [46]. Considering these factors, placental swabs may provide a better reflection of the intrauterine environment. Pathogens cultured on the foetal side of the placenta are often correlated with early-onset neonatal sepsis severity [47]. Certainly, placental swab culture findings provide useful clinical information for neonatologists to inform antibiotic therapy, especially where blood cultures are sterile [32].

While the clinical value of placental swab cultures for early-onset neonatal sepsis is well established, there is less evidence regarding maternal outcomes. Clinical factors such as antibiotic administration for suspected or confirmed maternal sepsis as well as antibiotic PPROM prophylaxis can confound culture findings and may account for the high rate of sterile blood cultures in our results. The high number of potential pathogens (165/430) from our placental culture samples suggests that peripartum antibiotics or PPROM antibiotic prophylaxis may have had less of an influence on our placental culture findings. Only women meeting the criteria for sepsis were included in this study, so it is likely that broad-spectrum antibiotics were administered at some point within their patient journey (antimicrobial details were not available, see limitations). A 2008 study evaluating the use of peripartum antibiotics and placental cultures found just 4.6% (n = 12/259) of placental cultures demonstrated positive results [48]. From this sample 42% (n = 5/12) received peripartum antibiotics. Although these findings differ from our results, disparities in sample techniques, different microbiological protocols and the fastidious nature of organisms causing infection are potential confounding variables. However, when infection persists or clinical deterioration is evident, this may be significant for both mother and newborn infant. The isolation of bacteria from placental cultures may guide appropriate antibiotic selection and the duration of treatment. Collectively, such a targeted and vigilant antibiotic usage would assist in reducing the emerging concern of perinatal antimicrobial resistance.

Bacteria can ascend from the genital tract to colonise the intrauterine cavity or spread haematogenously, infiltrating the placenta, decidua and intrauterine space [49]. Consequently, the transmission mode may influence both placental histology and swab cultures. The current method of placental swab culturing is considered highly specific but lacks sensitivity [48]. There are several techniques for placental swab culturing. Although there is no published evidence recommending one technique, it is essential that the external placental surface is not sampled instead of the subamniotic side as vaginal microorganisms can contaminate the sample [50]. In our institution, swab cultures of the foetal side of the placenta and the interface between the chorion and the amnion are taken. This method is simple and reproducible, while avoiding contaminants from the vaginal flora. Several studies incorporating this technique found similar sensitivities and specificities in identification of intrauterine infection suggesting a standardised technique is not only reproducible but may provide clinically useful information [46, 48, 51].

Intrapartum fever is an indication to perform a septic screen and is often associated with adverse perinatal outcomes [47]. Our study found more pathogens were detected in those who had instrumental and vaginal deliveries, indicating that mode of delivery may influence the risk of developing sepsis. Intrapartum fever and positive placental blood cultures are significantly associated with adverse perinatal outcomes including labour dystocia and emergency caesarean section [52]. However, it is unclear whether intra-partum fever or intrauterine infection leads to labour dystocia, or if labour dystocia is a risk factor for fever, intrauterine infection, or both [52]. Causes of these peripartum complications are usually multifactorial [53, 54].

Positive placental cultures however, irrespective of intrapartum fever, are associated with an increased risk for neonatal morbidity, thereby, obtaining placental cultures at delivery may assist in better risk assessment and management [52]. Our study demonstrated that placental swabs collected at caesarean section were more likely to be sterile than swabs obtained at vaginal delivery, indicating that contamination may occur in some circumstances. However in many cases the pathogens detected from placental swabs were not present in concurrent vaginal swabs, indicating that these detections should not be dismissed as contaminants. We believe this demonstrates reasonably that placenta swab cultures may have a role in identifying potential pathogens irrespective of the mode of delivery. The culture results from placenta swabs of “query sepsis” patients and non-“query sepsis” patients showed no statistical difference among the bacteria detected however, which undermines the predictive value of potential pathogens in the former group.

Chorioamnionitis is associated with significant morbidity and mortality for both mother and infant. Although the clinical presentation of chorioamnionitis is often heterogenous, the condition is most frequently diagnosed on histopathological examination [55]. While histological examination does not guarantee that inflammation is caused by an infective process, correlation between bacteriological and histological placental findings from women with suspected or proven chorioamnionitis has 70% concordance [56]. The presence of histological chorioamnionitis is sensitive, but not specific to diagnosis of intraamniotic infection [51]. Although our study did not consider placental histopathological findings, it is possible that discordant histology and bacteriology may exist. Several reasons may explain these variations: early bacterial infection before development of neutrophilic inflammatory response, antibiotic prophylaxis, fastidious bacteria, and histological sampling errors [56]. Furthermore, non-infective causes of inflammation including foetal hypoxia, amniotic fluid PH changes and presence of meconium may be potential confounders influencing histopathological findings [48]. The clinical context of each delivery should be accounted for, considering antenatal, intrapartum and postnatal factors during each histopathological examination for retrospective diagnosis to inform future management and counselling.

GAS (Group A Strep) was not detected from any blood cultures, placental swabs or vaginal swabs in our study. This organism has been reported as the cause of more than 50% of “very severe” peripartum sepsis [57], however the overall incidence of peripartum GAS disease is low [58]. The most recent summary statistics for invasive GAS (iGAS) disease in Ireland [59] reported 136 cases for all patients (no data for peripartum patients specifically) in Ireland in 2018 (age range 0–94 years, 55% male). This suggests that iGAS incidence in Ireland was low at the time of our study. An increased incidence of iGAS was reported in June 2023 [60] for the period since October 2022 (305 cases in five months), but children accounted for the biggest share of this increase.

On review of international guidelines [29–31], two of them [29, 31] recommend placental swabs as potential specimens for microbiological analysis, but do not give guidance as to what circumstances merit their use, apart from “as clinically indicated”(SOMANZ) and “guided by clinical suspicion of focus of infection” (RCOG). The most recent set of Irish guidelines [25] does not make reference to microbiological analysis, in contrast to previous versions of the document [27] which listed clinical specimens (“Appropriate cultures, e.g. blood, wound, vaginal swab, urine etc,”), but did not mention placental swabs. No Microbiology or Infectious Disease specialists were named on the guideline development group for this document, and no Microbiology or Infectious Diseases societies were listed in the groups chosen for consultation. A review of the published literature was also performed, and five papers discussing the diagnosis of maternal sepsis were selected and reviewed [10, 13, 21–23]: Four of these studies provided specific recommendations for microbiology specimens for diagnosis. All four studies recommended blood cultures, one as an exclusive culture test [21], another recommended a vaginal swab [10], one discussed body fluids such as urine and cerebrospinal fluid [23], and one suggesting swabs of the uterine cavity, wounds, and the cervix [22]. None mentioned placental swabs. The paucity of guidance and research in this area supports the need for further studies to determine the significance of placental swabs. This study adds to the body of evidence supporting the use of placental swabs in the management of perinatal sepsis.

Our study has several strengths and limitations. Strengths include the large dataset and uniform data collection. Placental swabs are non-invasive, reproducible and may provide clinically useful information. However, only patients meeting the criteria for maternal sepsis were included in this analysis, so our findings cannot be generalised beyond this cohort. The classification of patients as “query sepsis” was determined based on the clinical details submitted with their test requests (without access to order communications systems), this may not be a reliable method of identifying patients with suspected sepsis. Furthermore, the discretionary and subjective nature of decisions to request tests such as placental swabs and other "septic screen" specimens may have introduced bias into our findings. Due to the absence of electronic patient records, clinical information including patient outcomes, timing and the number of antibiotic dosages, vital signs, patient risk factors and medical histories were not available. The time taken for culture and susceptibility results to become available was not evaluated for the microbiology specimens in this study. Lastly, our examination of placental and vaginal cultures excluded both *Ureaplasma urealyticum* and *Mycoplasma hominis*. These two bacteria are detected commonly in chorioamnionitis (47% and 30% respective [61]), but are not known to be causes of maternal sepsis. Irrespective of these limitations, we believe our study has clinical merit and provides a useful descriptive analysis of potential pathogens implicated in maternal sepsis. Future studies may correlate and evaluate placental swab cultures containing likely pathogens with placental histology and clinical parameters.

## Conclusion

Placental swab cultures, collected as part of maternal septic screens, detected the highest number of potential pathogens, which may offer clinically useful information. Given 95% of blood cultures were sterile and just 2.4% contained organisms deemed to be pathogens, these specimens taken in isolation would have been insufficient to inform antimicrobial treatment for the majority of patients. The low positivity of blood cultures in our patients could have been influenced by insufficient cultures performed; just 2% (10/430) of our patients had the recommended [62] six or more blood culture bottles (ie three “sets”) tested for the investigation of sepsis. Throat swabs were not shown to be useful specimens for the screening of potential pathogens for the patients in our study. Throat swabs had low sensitivity as a screening tool for potential pathogens in our study. Given the knowledge that intra-uterine infections tend to be polymicrobial, the results from our placental swabs (mostly polymicrobial also) most closely matched that scenario. While high vaginal swabs also detected a high number of potential pathogens, they are more susceptible to contamination by vaginal flora. Placental swabs are easy to collect, process and are reproducible if completed using a standardised method. While contamination may affect culture results, our study shows that placental swab cultures may be clinically useful to detect significant isolates, particularly enterobacterales. The predictive value of the specimens in this study cannot be ascertained without access to the clinical context of the patients. Future studies could usefully evaluate whether placental swab cultures correlate with clinical parameters to establish their true clinical and prognostic value. The use of standardised protocols and/or multicentre evaluation would enhance consistency of reporting and provide more robust results. We recommend the input from microbiology and infectious disease specialists in the development of guidance regarding the management of perinatal sepsis.

## Supporting information

S1 Table Microbiology specimen recommendations (if clinically indicated) for the investigation of maternal sepsis, from clinical guidance documents available nationally and internationally. RCOG: Royal College of Obstetricians and Gynaecologists (UK); SMFM: Society for Maternal-Fetal Medicine (USA); SOMANZ: Society of Obstetric Medicine Australia and New Zealand; IMEWS: Irish Maternity Early Warning System (Ireland).
(DOCX)

S1 File Supplementary microbiology methods. (DOCX)

S2 File Supplementary material: Antimicrobial prescribing guidelines. (DOCX)

## References

[1] Padilla C, Palanisamy A. Managing maternal sepsis: early warning criteria to ECMO. Clin Obstet Gynecol. 2017;60(2):418–24. doi: 10.1097/GRF.0000000000000269 28098573

[2] UNICEF. Maternal mortality rate [Available from: https://data.unicef.org/topic/maternal-health/maternal-mortality/.

[3] Nair S, Spring A, Dockrell L, Mac Colgain S. Irish Maternal Early Warning Score. Ir J Med Sci. 2020;189(1):229–35. doi: 10.1007/s11845-019-02028-1 31254160

[4] Burlinson CEG, Sirounis D, Walley KR, Chau A. Sepsis in pregnancy and the puerperium. Int J Obstet Anesth. 2018;36:96–107. doi: 10.1016/j.ijoa.2018.04.010 29921485

[5] Escobar MF, Echavarría MP, Zambrano MA, Ramos I, Kusanovic JP. Maternal sepsis. Am J Obstet Gynecol MFM. 2020;2(3):100149. doi: 10.1016/j.ajogmf.2020.100149 33345880

[6] Frequency and management of maternal infection in health facilities in 52 countries (GLOSS): a 1-week inception cohort study. Lancet Glob Health. 2020;8(5):e661–e71. doi: 10.1016/S2214-109X(20)30109-1 32353314 PMC7196885

[7] Delano MJ, Ward PA. The immune system’s role in sepsis progression, resolution, and long-term outcome. Immunol Rev. 2016;274(1):330–53. doi: 10.1111/imr.12499 27782333 PMC5111634

[8] World Health Organisation. Statement on Maternal Sepsis 2017 [Available from: https://apps.who.int/iris/bitstream/handle/10665/254608/WHO-RHR-17.02-eng.pdf;sequence=1.

[9] Chakraborty RK BB. Systemic Inflammatory Response Syndrome: Treasure Island (FL): StatPearls Publishing; [Available from: https://www.ncbi.nlm.nih.gov/books/NBK547669/. 31613449

[10] Turner MJ. Maternal sepsis is an evolving challenge. Int J Gynaecol Obstet. 2019;146(1):39–42. doi: 10.1002/ijgo.12833 31037723

[11] Acosta CD, Kurinczuk JJ, Lucas DN, Tuffnell DJ, Sellers S, Knight M. Severe maternal sepsis in the UK, 2011–2012: a national case-control study. PLoS Med. 2014;11(7):e1001672. doi: 10.1371/journal.pmed.1001672 25003759 PMC4086731

[12] Peng CC, Chang JH, Lin HY, Cheng PJ, Su BH. Intrauterine inflammation, infection, or both (Triple I): A new concept for chorioamnionitis. Pediatr Neonatol. 2018;59(3):231–7. doi: 10.1016/j.pedneo.2017.09.001 29066072

[13] Bauer ME, Housey M, Bauer ST, Behrmann S, Chau A, Clancy C, et al. Risk Factors, Etiologies, and Screening Tools for Sepsis in Pregnant Women: A Multicenter Case-Control Study. Anesth Analg. 2019;129(6):1613–20. doi: 10.1213/ANE.0000000000003709 31743182 PMC7543988

[14] Megli CJ, Coyne CB. Infections at the maternal-fetal interface: an overview of pathogenesis and defence. Nat Rev Microbiol. 2022;20(2):67–82. doi: 10.1038/s41579-021-00610-y 34433930 PMC8386341

[15] Ely J W., Rijhsinghani A C. Bowdler N, D. Dawson J. The association between manual removal of the placenta and postpartum endometritis following vaginal delivery. Obstetrics & Gynecology. 1995;86(6):1002–6. 10.1016/0029-7844(95)00327-n 7501321

[16] Furman B, Shoham-Vardi I, Bashiri A, Erez O, Mazor M. Clinical significance and outcome of preterm prelabor rupture of membranes: population-based study. European Journal of Obstetrics & Gynecology and Reproductive Biology. 2000;92(2):209–16. 10.1016/s0301-2115(99)00257-2 10996683

[17] American College of Obstetricians and Gynecologists’ Committee on Obstetric Practice. Committee Opinion No. 712: Intrapartum Management of Intraamniotic Infection. Obstet Gynecol. 2017;130(2):e95–e101. 10.1097/AOG.0000000000002236 28742677

[18] Gilson GJ, Christensen F, Romero H, Bekes K, Silva L, Qualls CR. Prevention of group B streptococcus early-onset neonatal sepsis: comparison of the Center for Disease Control and prevention screening-based protocol to a risk-based protocol in infants at greater than 37 weeks’ gestation. J Perinatol. 2000;20(8 Pt 1):491–5. doi: 10.1038/sj.jp.7200463 11190588

[19] Kim JS, Kim YJ, Kim WY. Characteristics and clinical outcomes of culture-negative and culture-positive septic shock: a single-center retrospective cohort study. Crit Care. 2021;25(1):11. doi: 10.1186/s13054-020-03421-4 33407768 PMC7787242

[20] Li Y, Guo J, Yang H, Li H, Shen Y, Zhang D. Comparison of culture-negative and culture-positive sepsis or septic shock: a systematic review and meta-analysis. Crit Care. 2021;25(1):167. doi: 10.1186/s13054-021-03592-8 33964934 PMC8106121

[21] Foeller ME, Gibbs RS. Maternal sepsis: new concepts, new practices. Curr Opin Obstet Gynecol. 2019;31(2):90–6. doi: 10.1097/GCO.0000000000000523 30789841

[22] Adorno M. Sepsis in the Obstetric Client. Crit Care Nurs Clin North Am. 2018;30(3):415–22. doi: 10.1016/j.cnc.2018.05.012 30098745

[23] Chun K-m, Syndergaard C, Damas CEP, Trubey RK, Mukindaraj A, Qian S, et al. Sepsis Pathogen Identification. Journal of Laboratory Automation. 2015;20:539–61. doi: 10.1177/2211068214567345 25631157

[24] Pakyz AL, Orndahl CM, Johns A, Harless DW, Morgan DJ, Bearman GM, et al. Impact of the Centers for Medicare and Medicaid Services Sepsis Core Measure on Antibiotic Use. Clinical infectious diseases: an official publication of the Infectious Diseases Society of America. 2020. 10.1093/cid/ciaa456 32827032

[25] The Childbirth Guideline Development Group (GDG) of the HSE National Clinical Programme for Obstetrics and Gynaecology. Irish Maternity Early Warning System (IMEWS) V2 2019 [Available from: https://assets.gov.ie/35807/6f4f5017fd7a466bbb1b77041b19a03c.%204%20Full%20Report.

[26] HSE. Sepsis predisposition & recognition 2018 [Available from: https://www.hse.ie/eng/about/who/cspd/ncps/sepsis/resources/maternity-sepsis-form-2020.pdf.

[27] Institute of Obstetricians and Gynaecologists; Royal College of Physicians of Ireland; Directorate of Clinical Strategy and Programmes of the Health Service Executive. The Irish Maternity Early Warning System (IMEWS) 2014 [Available from: https://www.hse.ie/eng/services/publications/clinical-strategy-and-programmes/imews-guidelines.pdf.

[28] Maguire PJ, O’Higgins A, Power K, Turner MJ. The Irish Maternity Early Warning System (IMEWS). Irish Medical Journal. 2014;Nov-Dec;107(10):309. 25551897

[29] Royal College of Obstetricians and Gynaecologists. Bacterial Sepsis following Pregnancy (Green-top Guideline No. 64b) 2012 [Available from: https://www.rcog.org.uk/guidance/browse-all-guidance/green-top-guidelines/bacterial-sepsis-following-pregnancy-green-top-guideline-no-64b/.

[30] Plante LA, Pacheco LD, Louis JM. SMFM Consult Series #47: Sepsis during pregnancy and the puerperium. Am J Obstet Gynecol. 2019;220(4):B2–b10. doi: 10.1016/j.ajog.2019.01.216 30684460

[31] Bowyer L, Robinson HL, Barrett H, Crozier TM, Giles M, Idel I, et al. SOMANZ guidelines for the investigation and management sepsis in pregnancy. Aust N Z J Obstet Gynaecol. 2017;57(5):540–51. doi: 10.1111/ajo.12646 28670748

[32] Garvey A, Powell J, Murphy B, O’Connell N, Imcha M, Philip RK. Youngest survivor of perinatal infection by Eikenella corrodens: case analysis and literature review highlighting the merits of placental swab culture. New Microbes New Infect. 2018;21:81–5. doi: 10.1016/j.nmni.2017.10.012 29263790 PMC5726748

[33] Warren S, Tristram S, Bradbury RS. Maternal and neonatal sepsis caused by Haemophilus influenzae type d. J Med Microbiol. 2010;59(Pt 3):370–2. doi: 10.1099/jmm.0.016543-0 19926730

[34] Roll C, Schmid EN, Menken U, Hanssler L. Fatal Salmonella enteritidis sepsis acquired prenatally in a premature infant. Obstet Gynecol. 1996;88(4 Pt 2):692–3. doi: 10.1016/0029-7844(96)00076-2 8841255

[35] Arora P, Bagga R, Kalra J, Kumar P, Radhika S, Gautam V. Mean gestation at delivery and histological chorioamnionitis correlates with early-onset neonatal sepsis following expectant management in pPROM. J Obstet Gynaecol. 2015;35(3):235–40. doi: 10.3109/01443615.2014.958143 25244519

[36] Monari F, Gabrielli L, Gargano G, Annessi E, Ferrari F, Rivasi F, et al. Fetal bacterial infections in antepartum stillbirth: a case series. Early Hum Dev. 2013;89(12):1049–54. doi: 10.1016/j.earlhumdev.2013.08.010 24041816

[37] Neylon O, O’Connell NH, Slevin B, Powell J, Monahan R, Boyle L, et al. Neonatal staphylococcal scalded skin syndrome: clinical and outbreak containment review. Eur J Pediatr. 2010;169(12):1503–9. doi: 10.1007/s00431-010-1252-1 20625909

[38] O’Connor C, Philip RK, Kelleher J, Powell J, O’Gorman A, Slevin B, et al. The first occurrence of a CTX-M ESBL-producing Escherichia coli outbreak mediated by mother to neonate transmission in an Irish neonatal intensive care unit. BMC Infect Dis. 2017;17(1):16. doi: 10.1186/s12879-016-2142-6 28056822 PMC5217319

[39] O’Connor C, Powell J, Finnegan C, O’Gorman A, Barrett S, Hopkins KL, et al. Incidence, management and outcomes of the first cfr-mediated linezolid-resistant Staphylococcus epidermidis outbreak in a tertiary referral centre in the Republic of Ireland. J Hosp Infect. 2015;90(4):316–21. doi: 10.1016/j.jhin.2014.12.013 25648941

[40] O’Connor C, Cormican M, Boo TW, McGrath E, Slevin B, O’Gorman A, et al. An Irish outbreak of New Delhi metallo-β-lactamase (NDM)-1 carbapenemase-producing Enterobacteriaceae: increasing but unrecognized prevalence. J Hosp Infect. 2016;94(4):351–7. 27624807 10.1016/j.jhin.2016.08.005

[41] Huggard D, Powell J, Kirkham C, Power L, O’Connell NH, Philip RK. Time to positivity (TTP) of neonatal blood cultures: a trend analysis over a decade from Ireland. J Matern Fetal Neonatal Med. 2021;34(5):780–6. doi: 10.1080/14767058.2019.1617687 31072183

[42] Saeed KB, Corcoran P, Greene RA. Incisional surgical site infection following cesarean section: A national retrospective cohort study. Eur J Obstet Gynecol Reprod Biol. 2019;240:256–60. doi: 10.1016/j.ejogrb.2019.07.020 31344664

[43] O’Loughlin R, Allwright S, Barry J, Kelly A, Teljeur C. Using HIPE data as a research and planning tool: limitations and opportunities. Ir J Med Sci. 2005;174(2):40–5; discussion 52–7. doi: 10.1007/BF03169128 16094912

[44] Beasley TM, Schumacker RE. Multiple regression approach to analyzing contingency tables: Post hoc and planned comparison procedures. The Journal of Experimental Education. 1995;64(1):79–93.

[45] Verani JR, McGee L, Schrag SJ. Prevention of perinatal group B streptococcal disease—revised guidelines from CDC, 2010. MMWR Recomm Rep. 2010;59(Rr-10):1–36. 21088663

[46] Kunze M, Zubler B, Müller C, Flecken U, Clad A. Evaluation of placental membrane swabs in the diagnosis of intrauterine infections. Geburtshilfe und Frauenheilkunde. 2006;66(06):575–8.

[47] Berger A, Witt A, Haiden N, Kretzer V, Heinze G, Pollak A. Amniotic cavity cultures, blood cultures, and surface swabs in preterm infants–useful tools for the management of early-onset sepsis? 2004;32(5):446–52. 10.1515/JPM.2004.145 15493724

[48] BHOLA K AL-KINDI H, FADIA M, KENT AL, COLLIGNON P, DAHLSTROM JE. Placental cultures in the era of peripartum antibiotic use. Australian and New Zealand Journal of Obstetrics and Gynaecology. 2008;48(2):179–84. 18366492 10.1111/j.1479-828X.2008.00833.x

[49] Redline RW, editor Placental inflammation. Seminars in Neonatology; 2004: Elsevier. 10.1016/j.siny.2003.09.005 15251143

[50] Aquino TI, Zhang J, Kraus FT, Knefel R, Taff T. Subchorionic fibrin cultures for bacteriologic study of the placenta. Am J Clin Pathol. 1984;81(4):482–6. doi: 10.1093/ajcp/81.4.482 6702749

[51] Pettker CM, Buhimschi IA, Magloire LK, Sfakianaki AK, Hamar BD, Buhimschi CS. Value of placental microbial evaluation in diagnosing intra-amniotic infection. Obstetrics & Gynecology. 2007;109(3):739–49. doi: 10.1097/01.AOG.0000255663.47512.23 17329528

[52] Ashwal E, Salman L, Tzur Y, Aviram A, Ben-Mayor Bashi T, Yogev Y, et al. Intrapartum fever and the risk for perinatal complications—the effect of fever duration and positive cultures. J Matern Fetal Neonatal Med. 2018;31(11):1418–25. doi: 10.1080/14767058.2017.1317740 28391772

[53] Thierrin L, Mercier FJ. Epidural analgesia and fever during labor. J Gynecol Obstet Biol Reprod (Paris). 2005;34(5):423–6. 16142132 10.1016/s0368-2315(05)82849-2

[54] Yancey MK, Zhang J, Schwarz J, Dietrich CS, 3rd, Klebanoff M. Labor epidural analgesia and intrapartum maternal hyperthermia. Obstet Gynecol. 2001;98(5 Pt 1):763–70. 11704166 10.1016/s0029-7844(01)01537-x

[55] Smulian JC, Shen-Schwarz S, Vintzileos AM, Lake MF, Ananth CV. Clinical chorioamnionitis and histologic placental inflammation. Obstet Gynecol. 1999;94(6):1000–5. doi: 10.1016/s0029-7844(99)00416-0 10576190

[56] Queiros da Mota V, Prodhom G, Yan P, Hohlfheld P, Greub G, Rouleau C. Correlation between placental bacterial culture results and histological chorioamnionitis: a prospective study on 376 placentas. J Clin Pathol. 2013;66(3):243–8. doi: 10.1136/jclinpath-2012-201124 23268318

[57] Sriskandan S. Severe peripartum sepsis. J R Coll Physicians Edinb. 2011;41(4):339–46. doi: 10.4997/JRCPE.2011.411 22184573

[58] Anderson BL. Puerperal group A streptococcal infection: beyond Semmelweis. Obstet Gynecol. 2014;123(4):874–82. doi: 10.1097/AOG.0000000000000175 24785617

[59] Health Protection and Surveillance Centre. Invasive Group A Streptococcal Disease in Ireland, 2018 2019 [Available from: https://www.hpsc.ie/a-z/other/groupastreptococcaldiseasegas/surveillancereports/.

[60] Protection Health and Centre Surveillance. Update on Group A streptococcus 2023 [Available from: https://www.hpsc.ie/a-z/other/groupastreptococcaldiseasegas/title-22663-en.html.

[61] Tita AT, Andrews WW. Diagnosis and management of clinical chorioamnionitis. Clin Perinatol. 2010;37(2):339–54. doi: 10.1016/j.clp.2010.02.003 20569811 PMC3008318

[62] Lamy B, Roy P, Carret G, Flandrois JP, Delignette-Muller ML. What is the relevance of obtaining multiple blood samples for culture? A comprehensive model to optimize the strategy for diagnosing bacteremia. Clin Infect Dis. 2002;35(7):842–50. doi: 10.1086/342383 12228821

