# Supplementary material for: The microbial pathology of maternal perinatal sepsis: A single-institution retrospective five-year review
Source: PLoS One. 2023 Dec 27;18(12):e0295210. doi: 10.1371/journal.pone.0295210 (PMC10752550; doi:10.1371/journal.pone.0295210)
Supplement: S1 Table — RCOG: Royal College of Obstetricians and Gynaecologists (UK); SMFM: Society for Maternal-Fetal Medicine (USA); SOMANZ: Society of Obstetric Medicine Australia and New Zealand; IMEWS: Irish Maternity Early Warning System (Ireland). (DOCX) [file pone.0295210.s001.docx]

| **RCOG(28)** | **SMFM(29)** | **SOMANZ(30)** | **IMEWS(24)** |
| --- | --- | --- | --- |
| Blood | Blood | Blood | Blood |
| Urine | Urine | Urine | Urine |
| Sputum | Sputum | Sputum | - |
| Wound swabs | - | Wound swabs | Wound swabs |
| CSF | - | CSF | - |
| Placenta swab | - | Placenta swab | - |
| Throat Swab | - | Naso-pharyngeal Swab | - |
| Vaginal swab | - | Vaginal swab | Vaginal swab |
| - | - | Stool culture | - |
| - | - | Amniotic Fluid | - |
| Breast Milk | - | - | - |
| Other samples as guided by clinical suspicion of focus of infection | Others as indicated | Additional sites as clinically indicated | Other appropriate cultures |

Supplementary Table 1. Microbiology specimen recommendations (if clinically indicated) for the investigation of maternal sepsis, from clinical guidance documents available nationally and internationally. RCOG: Royal College of Obstetricians and Gynaecologists (UK); SMFM: Society for Maternal-Fetal Medicine (USA); SOMANZ: Society of Obstetric Medicine Australia and New Zealand; IMEWS: Irish Maternity Early Warning System (Ireland).
