# Supplementary material for: The microbial pathology of maternal perinatal sepsis: A single-institution retrospective five-year review
Source: PLoS One. 2023 Dec 27;18(12):e0295210. doi: 10.1371/journal.pone.0295210 (PMC10752550; doi:10.1371/journal.pone.0295210)
Supplement: S1 File — (DOCX) [file pone.0295210.s002.docx]

Supplementary Microbiology Methods

Blood Cultures

5-10 mls (recommended) volume of blood per bottle were aseptically inoculated into paired BacT/ALERT® FAN® Plus aerobic and anaerobic blood culture bottles and incubated in the BacT/ALERT 3D automated microbial detection system for a maximum of five days. Positive cultures were monitored 24 hours per day, and sub-cultured onto solid agar depending on the Gram stain result. Isolates were identified using Bruker Microflex® MALDI-TOF spectrometry (Bruker PLC, Billerica, USA).

All microbiology plate cultures were performed on 90mm Petri dish pre-poured commercially supplied agar from LIP Diagnostics, Fannin Ltd, Galway, Ireland or BioMérieux PLC, Marcy-l'Étoile, France.

Susceptibility testing: Antibiotic susceptibility testing was performed for all isolates using Sensititre ARIS 2X, in accordance with the European Committee on Antimicrobial Susceptibility Testing (EUCAST) guidelines Version 12.0, 2022.

Throat Swabs

Throat swabs were collected using viscose (rayon) tipped swabs in Amies transport medium with charcoal (Deltalab, Barcelona, Spain) and were sub-cultured onto Staph/Strep agar (LIP), incubated anaerobically at 35-37^o^ C for 18-24 hours, checked for pathogens, re-incubated for a further 24 hours and checked again. Target organisms: Haemolytic *Streptococci* (principally *Streptococcus pyogenes*), *Arcanobacterium haemolyticum*. Other organisms reported if found: *Candida* species, *Staphylococcus aureus*, anaerobes.

Urines

Urine specimens were cultured onto CHROMID® CPS® Elite agar (BioMérieux) and incubated in an aerobic atmosphere 35-37^o^ C for 18-24 hours. Target organisms: Enterobacterales (principally *Escherichia coli*), *Enterococcus* species, *Staphylococcus aureus*, *Staphylococcus saprophyticus*, *Pseudomonas* species.

High Vaginal Swabs

High vaginal swabs (rayon tipped swabs in Amies transport medium with charcoal) from “query sepsis” patients were cultured onto Colombia blood agar (LIP), incubated in an atmosphere containing 5-10% CO_2_ at 35-37^o^ C for 48 hours, Colombia blood agar plus neomycin (LIP) incubated in an anaerobic atmosphere 35-37^o^ C for 48 hours, and Granada agar (BioMérieux) incubated in an anaerobic atmosphere 35-37^o^ C for 48 hours. Target organisms were *Staphylococcus aureus*, Beta-Haemolytic *Streptococcus* (principally *Streptococcus agalactiae*), *Streptococcus milleri*, *Candida* species, anaerobes, enterobacterales.

Placenta Swabs

Placenta swabs were collected at the discretion of clinicians when chorioamnionitis was suspected clinically or in cases of maternal pyrexia in labour, prolonged rupture of membranes (PROM), preterm prelabour rupture of membanes (PPROM) or if foul smelling liquor was noted. Swabbing was performed of the chorionic and the amniotic sides, using one swab (rayon tipped swabs in Amies transport medium with charcoal) for each side. Swabs were sub-cultured onto agar plates as per the protocol for “query sepsis” high vaginal swabs (see above), with the addition of Colombia chocolate agar (LIP) for fastidious organisms and *Listeria* selective agar for the detection of *Listeria monocytogenes*.
