# Supplementary material for: The microbial pathology of maternal perinatal sepsis: A single-institution retrospective five-year review
Source: PLoS One. 2023 Dec 27;18(12):e0295210. doi: 10.1371/journal.pone.0295210 (PMC10752550; doi:10.1371/journal.pone.0295210)
Supplement: S2 File — (DOCX) [file pone.0295210.s003.docx]

Supplementary Material: Antimicrobial Prescribing Guidelines

**GBS Prophylaxis/Intra-partum prophylaxis**

No known penicillin allergy:

Benzylpenicillin 3g as soon as possible after the onset of labour and 1.8g 4 hourly until delivery

Non –immediate penicillin allergy:

Cefuroxime 1.5g Six hourly IV as soon as possible after the onset of delivery until delivery

Severe or immediate pencillin allergy

If GBS is isolated as is known to be clindamycin susceptible, then use clindamycin 900mg tds IV as soon as possible after onset of labour until delivery

If GBS is isolated and is known to be resistant to Clindamycin or if susceptibility to clindamycin is unknown use Vancomycin 1g 12 hourly IV (max 2g) as soon as possible after onset of labour until delivery

**PPROM prophylaxis**

Preterm pre-labour rupture of membranes

Erythromycin 250mg QDS x 10 days or until delivery.

Oral erythromycin is indicated as antibiotic prophylaxis only and is started following PPROM diagnosis from 20 weeks gestation as long as there is no clinical evidence of chorioamnionitis or maternal sepsis. Erythromycin is NOT appropriate if the woman is clinically unwell.

GBS sensitivity to erythromycin should be known or established – HVS swabs at PPROM should specifically request GBS sensitivity testing on the laboratory form.

**Antenatal sepsis**

This regimen is not suitable for known or suspected MDROs.

No known penicillin allergy:

Benzylpenicillin 2.4g QDS IV

+

Metronidazole 500mg IV tds

+

Gentamicin 5mg/kg 24 hourly (max 480mg)

Alt:

Co-amoxiclav 1.2g tds IV

+

Gentamicin (as above)

Early escalation to Piperacillin-tazobactam 4.5g tds IV and Gentamicin may be warranted depending on clinical severity, recent microbiology results or recent use of co-amoxiclav.

Non-immediate penicillin allergy

Cefuroxime 1.5g qds and metronidazole and Gentamicin

Severe or immediate penicillin allergy

If GBS isolated and known Clindamycin susceptible

Clindamycin and gentamicin.

If GBS isolated and is known to be clindamycin resistant or if susceptibility is unknown use vancomycin and gentamicin.

Note:

In septic shock may consider escalation to include meropenem

**POST NATAL sepsis**

No known penicillin allergy

Co-amoxiclav 1.2g Tds and gentamicin 5mg./kg od

Early escalation to piperacillin-tazobactam and gentamicin

Non immediate penicillin allergy

Cefuroxime and metronidazole and gentamicin

Severe or immediate pen allergy

If GBS isolated and known to be Clindamycin S

Clindamycin and gent

If GBS isolated and known to be clindamycin R or not known

Vancomycin and gent

Escalated in septic shock

No allergy: Mero and gent and clindamycin and vancomycin if risk MRSA

Non immediate: meropenem and gent and clindamycin and vancomycin if risk MRSA

Severe/immediate: vancomycin and gentamicin + Ciprofloxacin or meropenem on case by case basis.
